# Hydrogen Sulfide in the Adipose Tissue—Physiology, Pathology and a Target for Pharmacotherapy

**DOI:** 10.3390/molecules22010063

**Published:** 2016-12-31

**Authors:** Jerzy Bełtowski, Anna Jamroz-Wiśniewska

**Affiliations:** 1 Department of Pathophysiology, Medical University, 20-090 Lublin, Poland; 2 Department of Neurology, Medical University, 20-090 Lublin, Poland

**Keywords:** hydrogen sulfide, adipose tissue, lipolysis, insulin resistance, adipogenesis, vascular tone, obesity, metabolic syndrome

## Abstract

Hydrogen sulfide (H_2_S) is synthesized in the adipose tissue mainly by cystathionine γ-lyase (CSE). Several studies have demonstrated that H_2_S is involved in adipogenesis, that is the differentiation of preadipocytes to adipocytes, most likely by inhibiting phosphodiesterases and increasing cyclic AMP concentration. The effect of H_2_S on adipose tissue insulin sensitivity and glucose uptake is controversial. Some studies suggest that H_2_S inhibits insulin-induced glucose uptake and that excess of H_2_S contributes to adipose tissue insulin resistance in metabolic syndrome. In contrast, other studies have demonstrated that H_2_S stimulates glucose uptake and its deficiency contributes to insulin resistance. Similarly, the effect of H_2_S on adipose tissue lipolysis is controversial. H_2_S produced by perivascular adipose tissue decreases vascular tone by activating ATP-sensitive and/or voltage-gated potassium channels in smooth muscle cells. Experimental obesity induced by high calorie diet has a time dependent effect on H_2_S in perivascular adipose tissue; short and long-term obesity increase and decrease H_2_S production, respectively. Hyperglycemia has been consistently demonstrated to suppress CSE-H_2_S pathway in various adipose tissue depots. Finally, H_2_S deficiency may contribute to adipose tissue inflammation associated with obesity/metabolic syndrome.

## 1. Introduction

Studies performed during the last two decades indicate that endogenous hydrogen sulfide (H_2_S) plays an important role in the regulation of many physiological processes such as neurotransmission, vascular tone, inflammatory and immune reactions, gastrointestinal function, cancer development, etc. [1,2,3,4,5]. H_2_S is synthesized from l-cysteine and/or l-homocysteine in at least three enzymatic pathways catalyzed by cystathionine β-synthase (CBS), cystathionine gamma-lyase (CSE) and cysteine aminotransferase together with 3-mercaptopyruvate sulfurtransferase (3-MST) [6]. To exert its biological activities, H_2_S exploits several unique molecular signaling mechanisms such as sulfidation of protein thiol (-SH) to persulfide (-SSH) groups, reaction with reactive oxygen (e.g., superoxide anion radical, hydrogen peroxide, etc.) and nitrogen (nitric oxide, NO) species, and interaction with hemeproteins [7,8,9]. In addition, H_2_S is actively metabolized in mitochondria by several consecutively acting enzymes: sulfide:quinone oxidoreductase (SQR), thiosulfate:cyanide sulfurtransferase (TST, rhodanese), persulfide dioxygenase and sulfite oxidase with thiosulfate (SSO_3_^2−^) and sulfate (SO_4_^2−^) being the final products [10,11].

The role of H_2_S in the regulation of cardiovascular, gastrointestinal, central and peripheral nervous system function as well as in the regulation of inflammatory and immune response has been described in many excellent recent review articles [1,2,3,4,5,6]. In this paper we will briefly review the role of H_2_S in adipose tissue which was, until now, much less studied, although during the last 10 years some important data have been accumulated in this area.

## 2. Adipose Tissue—An Overview

Adipose tissue is quantitatively one of the most abundant tissues in the human body, however, until recently it was quite neglected and not extensively studied. For a long time, adipose tissue was considered only as the passive site of energy storage. There are two major types of the adipose tissue: white adipose tissue (WAT) and brown adipose tissue (BAT). Adipose tissue consists of adipocytes—highly specialized cells able to take up and accumulate large amounts of triglycerides during periods of energy excess and to mobilize them under conditions of energy deficiency [12,13]. In addition, adipose tissue contains a stromovascular fraction consisting of blood and lymphatic vessels, fibroblasts, preadipocytes and inflammatory and immune cells such as macrophage and lymphocytes. White adipocytes contain a big lipid droplets consisting of triglycerides. Lipoprotein lipase (LPL)—the enzyme attached to luminal surface of endothelial cells—hydrolyzes triglycerides contained in plasma lipoproteins and releases fatty acids which are then taken up and esterified inside the adipocyte. In this way, adipocytes accumulate fatty acids obtained from alimentary sources and synthesized in the liver which are transported in the blood by chylomicrons and very low density lipoproteins, respectively. In addition, fatty acids are synthesized de novo inside the adipocytes from acetyl-coenzyme A, the product of glucose metabolism. Triglycerides stored in lipid droplets are substrates for lipolysis—the complex and highly regulated process catalyzed by three consecutively acting enzymes, adipocyte triglyceride lipase (ATGL), diglyceride lipase (hormone sensitive lipase, HSL) and monoglyceride lipase (MGL) [14,15,16]. The final products of lipolysis are glycerol and non-esterified fatty acids (NEFA). Glycerol is released to the extracellular space and then is metabolized by the liver whereas NEFA may be either released or re-esterified in adipocytes to triglycerides with glycerol 3-phosphate provided by glycolysis as the co-substrate. NEFA released from the adipose tissue represent the energy substrate for other tissues such as the skeletal muscles and the heart as well as may be partially oxidized to ketone bodies in the liver.

Adipose tissue lipolysis is a highly regulated process [17,18]. Hormone sensitive lipase is phosphorylated and stimulated by cyclic adenosine monophosphate (cAMP)-activated protein kinase A (PKA). Both circulating catecholamines and noradrenaline released by the sympathetic endings within the adipose tissue stimulate β_2_ and/or β_3_ adrenergic receptors on the surface of adipocytes, increase intracellular cAMP and stimulate lipolysis. In addition, some other G-protein coupled receptors are expressed in adipocytes which can stimulate adenylate cyclase. Cyclic GMP (cGMP)-sensitive protein kinase G (PKG) may also phosphorylate and activate HSL; this pathway is stimulated by factors such as cardiac natriuretic peptides and nitric oxide which activate membrane-bound or soluble guanylyl cyclase, respectively. The main factor which inhibits lipolysis is insulin which decreases intracellular cAMP concentration by activating phosphodiesterases. In addition, insulin stimulates glucose uptake by the adipocytes, fatty acid synthesis and esterification leading to triglyceride accumulation. Indeed, adipose tissue is one of the main insulin target tissues and to large extent contributes to the whole-body insulin sensitivity.

During the last two decades it became apparent that adipose tissue is also the active endocrine organ [19,20]. In 1994 the first adipose tissue hormone, leptin, was identified and characterized. Leptin is produced by WAT in amounts proportional to energy stores and acts on hypothalamic centers to inhibit food intake and increase energy expenditure. However, leptin receptors are expressed not only in the brain but also in many peripheral tissues. Since that time a lot of other adipose tissue-derived protein hormones or “adipokines” have been identified. Some of them such as leptin, adiponectin or resistin are specific for adipose tissue. In addition, adipose tissue produces classic cytokines (tumor necrosis factor-α, TNF-α, interleukin-6), chemokines (interleukin-8, monocyte chemoattractant protein-1, MCP-1), hormones (angiotensin II, adrenomedullin, atrial natriuretic peptide), growth factors, enzymes and enzyme inhibitors (visfatin, vaspin, plasminogen activator inhibitor-1) and acute phase proteins. Adipose tissue secretes also non-protein biologically active mediators including gasotransmitters (NO, CO and H_2_S) [21,22,23,24,25].

Brown adipose tissue consists of specific brown adipocytes with small lipid droplets and high amount of mitochondria [26,27]. BAT is densely innervated by the sympathetic system and is characterized by continuous high-rate lipolysis. Most of non-esterified fatty acids in brown adipose tissue are oxidized locally in mitochondria in the uncoupled manner. Due to high expression of uncoupling protein-1 (UCP-1) electron transport in mitochondrial respiratory chain is not coupled to ATP synthesis and most of energy is dissipated as heat. Thus, BAT plays an important role in thermogenesis. Until recently, it was believed that BAT is present only in small animals whereas in humans and other large mammals it is confined to the early postnatal period. However, functional imaging studies have demonstrated that BAT deposits are active life-long in humans and their amount and activity are inversely correlated with total body adiposity [28,29]. In rodents such as rats and mice the largest BAT depot is located in the interscapular region and smaller depots are found close to blood vessels in the cervical, axillary and paravertebral regions as well as in the proximity of thoracic and bdominal viscera. In humans interscapular BAT disappears soon after infancy, however, in adults BAT depots are found within the subcutaneous WAT of the neck, thorax and abdomen as well as close to deep viscera (heart, lungs, kidney, adrenals, intestine) and along the large blood vessels [30]. In addition, brown adipocytes may appear within WAT, especially subcutaneous WAT, throughout life; the process referred to as “WAT browning” [31]. These new brown adipocytes are called brite (brown-in-white) or beige fat cells [32]. Activity of existing BAT depots as well as WAT browning are stimulated by factors such as cold exposure, exercise, stimulation of sympathetic nervous system, thyroid hormones as well as several mediators including fibroblast growth factor-21 (FGF21), β-aminoisobutyric acid, bile acids, adenosine, melatonin and natriuretic peptides [33,34,35,36].

Growing interest in adipose tissue results to large extent from markedly increasing prevalence of obesity and the related co-morbidities such as hyperlipidemia, impaired glucose tolerance/type 2 diabetes and arterial hypertension together referred to as metabolic syndrome. Although the pathogenesis of metabolic syndrome was originally attributed to insulin resistance and compensatory hyperinsulinemia, other factors such as dysregulation of adipokines (that is increase in secretion of some of them such as leptin and deficiency of others such as adiponectin) play also an important role. In particular, metabolic syndrome is associated with chronic low-grade inflammation of the adipose tissue characterized by accumulation of proinflammatory macrophages and lymphocytes as well as overproduction of cyto- and chemokines which substantially contribute to the development of insulin resistance itself as well as metabolic syndrome comorbidities [37,38,39,40].

## 3. Sources and Regulation of H_2_S in the Adipose Tissue

Feng et al. [41] were the first to demonstrate that H_2_S is produced by epididymal, perirenal and brown adipose tissue of the rat. Both CBS and CSE were identified at the mRNA level in these adipose tissue depots, however, CSE inhibitors propargylglycine (PAG) or β-cyano-l-alanine (BCA) reduced H_2_S production by adipose tissue by more than 80% suggesting that CSE is the main H_2_S synthase. These findings have been confirmed later in other adipose tissue depots as well as in cultured adipocytes.

The expression of CSE and H_2_S production by rat epididymal and perirenal adipose tissue increased with age [41]. In contrast, increasing glucose concentration from physiological (5 mM) to hyperglycemic range (20 mM) reduced CSE expression and H_2_S production. The effect of glucose was not reproduced by 20 mM mannitol indicating that it did not result from hyperosmolality.

## 4. Role of H_2_S in the Regulation of Adipogenesis

Adipogenesis, that is the formation of new adipocytes from their precursors, is a highly regulated process which contributes markedly to the amount and properties of adipose tissue. Conversion of preadipocytes into adipocytes is characterized by the sequential activation of several transcription factors such as peroxisome proliferator-activated receptor-gamma (PPAR-γ), CCAT enhancer binding protein-α (C/EBP α), sterol regulatory element-binding protein-1 (SREBP1) and carbohydrate response element-binding protein (ChREBP) [42,43]. Recently, Tsai et al. [44] examined the role of H_2_S in the regulation of adipogenesis using a fibroblast-like mouse preadipocyte cell line, 3T3-L1. Stimulation of adipogenesis with the mixture of insulin, dexamethasone and PDE inhibitor, isobutylmethylxanthine (IBMX) was associated with the increase in CBS, CSE and 3-MST expression in these cells as well as increase in H_2_S concentration in the culture medium. Treatment of 3T3-L1 preadipocytes with both inorganic (NaHS) or organic (GYY4137) H_2_S donors (both at 50 μM) stimulated the expression of PPARγ and C/EBPα (early markers of adipogenesis) at the mRNA level. In addition, GYY4137 but not NaHS increased the expression of ChREBP, SREBP1 and adipocyte-specific proteins: fatty acid-binding protein-4 (FABP4), perilipin A, hormone-sensitive lipase and fatty acid synthase (FAS). These effects of GYY4137 were mediated by H_2_S because were not reproduced by the related compound which does not release H_2_S, ZYJ1122. In contrast, CBS inhibitor, aminooxyacetic acid (AOAA) as well as CSE inhibitor, propargylglycine, applied during 3T3-L1 cell differentiation reduce the expression of C/EBPα, ChREBP, SREBP1, HSL and perilipin A. The similar effect was observed when either CBS or CSE were silenced by the specific small interfering RNAs. Moreover, NaHS or GYY4137 increased triglyceride accumulation (Oil Red staining) whereas AOAA and PAG had no effect on triglyceride accumulation in differentiating 3T3-L1 cells. Taken together, these results suggest that H_2_S production is up-regulated during adipogenesis and both endo- and exogenous H_2_S stimulates this process whereas inhibiting H_2_S suppresses adipogenesis.

Similarly, Cai et al. [45] have demonstrated that either H_2_S-saturated buffer (H_2_S concentration 200 μM), or GYY4137 (200 μM) added during differentiation of 3T3-L1 preadipocytes to the mature adipocytes increased lipid accumulation, perilipin 1 expression and glycerol 3-phosphate dehydrogenase activity in these cells. The same was observed in cells overexpressing CSE. In contrast, PAG or CSE knockdown by siRNA transfection reduced lipid accumulation, PPAR-γ and perilipin 1 expression, nuclear translocation of PPAR-γ and glycerol 3-phosphate dehydrogenase activity. The effect of H_2_S on adipogenesis may be mediated by inhibition of phosphodiesterase. Indeed, preadipocyte to adipocyte differentiation requires high intracellular cAMP concentration and most of culture media used to induce adipogenesis contain PDE inhibitor. GYY4137 at concentrations from 1 to 100 μM inhibited PDE activity in 3T3-L1 preadipocytes in a concentration-dependent manner whereas PAG increased it [46]. These data suggest that H_2_S—production of which increases during adipogenesis—suppresses PDE, increases cAMP concentration and stimulates adipocyte differentiation. Indeed, replacement of commonly used synthetic PDE inhibitor, IBMX, by H_2_S or GYY4137 in adipogenesis-stimulating coctail induced preadipocytes-to-adipocyte transformation [45].

These findings are consistent with the observation that both CBS knockout mice [47] and CSE knockout mice [48] are characterized by lower body weight and adipose tissue depots that wild-type animals and that homocystinuria (homozygous CBS deficiency) is associated with lipodystrophy in humans. In contrast, patients with Down syndrome bearing three copies of the CBS gene (localized in humans in chromose 21) are often obese.

In contrast, Ambati et al. [49] and Li et al. [50] have demonstrated that two garlic-derived organic sulfides, ajoene and diallyl trisulfide (DATS) which may release H_2_S in biological systems suppressed adipogenesis in cultured 3T3-L1 cells. DATS applied at 50–75 μM decreased the expression of C/EBPα, C/EBPβ, PPARγ and FAS in 3T3-L1 cells. In addition, DATS increased phosphorylation of extracellular signal regulated kinases (ERK), and ERK inhibitor, PD98059, abolished suppressing effect of DATS on adipogenesis. These results indicate that DATS inhibits adipogenesis in ERK-dependent manner. However, it is unclear if this effect of garlic polysulfides is mediated by H_2_S.

## 5. Role of H_2_S in the Regulation of Adipose Tissue Lipolysis

The role of H_2_S in the regulation of adipose tissue lipolysis is controversial. Geng et al. [46] have demonstrated that GYY4137 or l-cysteine reduced basal and isoproterenol-stimulated glycerol release by rat epididymal adipocytes which was accompanied by the decrease in HSL phosphorylation at Ser^639^ whereas CSE inhibitor, PAG, had the opposite effect. These findings suggest that H_2_S inhibits lipolysis. In vivo, PAG increased fasting glycerol level in normal diet-fed mice and tended, although not significantly, to increase in in high fat-fed mice. In contrast, GYY4137 reduced glycerol concentration in mice fed the high fat diet. In addition, high fat feeding for 13 weeks was associated with down-regulation of CSE-H_2_S system in adipose tissue.

Recently, we examined the role of H_2_S in the regulation of lipolysis using the microdialysis method. Under general anesthesia the microdialysis probe was inserted into the subcutaneous adipose tissue and was perfused with Ringer’s solution for 3 h. Dialysate was collected every 10 min and lipolysis was estimated by measuring glycerol level. The method is often used to assess adipose tissue metabolism because allows to monitor local tissue metabolism in vivo while maintaining systemic neurohormonal regulatory mechanisms. We observed that sodium sulfide (Na_2_S) added to the perfusate induced the concentration-dependent stimulation of glycerol release, although its effect was less potent than the effect of isoproterenol. The effect of Na_2_S was accompanied by cAMP release from the adipose tissue and was abolished by specific protein kinase A inhibitor, KT5720. In addition, we found that H_2_S release to the dialysate was markedly higher in rats fed the high-fat diet for 1 month than in control lean animals. As expected, glycerol release was greater in high fat diet-fed rats. Addition of PAG to the perfusate had no effect on glycerol release in control rats but partially reduced glycerol concentration in the prefusate of obese rats. These preliminary results suggest that H_2_S stimulates adipose tissue lipolysis in cAMP-PKA dependent manner. Moreover, up-regulation of CSE-H_2_S pathway in the adipose tissue may contribute to enhanced lipolysis in high fat diet fed obese animals.

## 6. Effect of H_2_S on Adipose Tissue Insulin Sensitivity and Glucose Uptake

Feng et al. [41] have demonstrated that H_2_S (10–1000 μM in solution) reduced basal and insulin-stimulated glucose uptake in primary culture of rat adipocytes. The similar was observed when endogenous H_2_S production was increased by adding l-cysteine and pyridoxal 5′-phosphate to the medium. In contrast, PAG or BCA increased basal and insulin-stimulated glucose uptake by 40% indicating that H_2_S produced under baseline conditions inhibits glucose uptake. The effect of H_2_S on glucose uptake was blocked by phosphoinositide 3-kinase inhibitor, LYY294002, but not by ATP-sensitive potassium channel inhibitor, glibenclamide. In addition, K_ATP_ channel agonist, pinacidil, had no effect on glucose uptake. These data suggest that K_ATP_ channels, one of the main targets for H_2_S in the vascular system, are not involved in the effect of H_2_S on adipocytes. The finding that suppressing effect of H_2_S on glucose uptake is mediated by PI3K is surprising because PI3K is the important component of insulin signaling mechanism.

Furthermore, Feng et al. [41] have demonstrated that feeding rats with high fructose diet, which is a commonly used model of insulin resistance and hyperlipidemia, increases CSE expression and H_2_S production in the adipose tissue which correlates with impaired insulin-induced glucose uptake. Together, these findings suggest that hyperactivity of the CSE-H_2_S system is involved in adipose tissue insulin resistance in the metabolic syndrome.

Tumor necrosis factor-α (TNF-α) is overproduced in the adipose tissue of obese animals and humans and contributes to the insulin resistance by targeting several components of the insulin signaling pathway [51]. Huang et al. [52] have demonstrated that TNF-α applied for 24 h inhibits insulin-induced glucose uptake by 3T3-L1 adipocytes and this effect is accompanied by the increase in CSE expression and activity as well as H_2_S accumulation in the medium. The detrimental effect of TNF-α on insulin-induced glucose uptake was attenuated by PAG or BCA but not by AOAA. In contrast to CSE inhibitors, NaHS at concentrations 100–1000 μM reduced insulin-induced glucose uptake and metabolism. Thus, TNF-α induced H_2_S overproduction may contribute to detrimental effect of this cytokine on adipose tissue insulin sensitivity.

In contrast, Cai et al. [45] have demonstrated that H_2_S solution or GYY4137 stimulated glucose uptake by 3T3-L1 adipocytes. This effect was associated with persulfidation of PPAR-γ at Cys^139^ and was abolished by dithiotreitol (DTT) known to reduce persulfide back to the thiol groups. Stimulatory effect of H_2_S on glucose uptake was not observed in adipocytes transfected with PPAR-γ mutant with Cys^139^ replaced by serine which cannot be persulfidated.

These findings are consistent with those of Manna et al. [53] who examined the effect of H_2_S on insulin sensitivity in 3T3-L1 adipocytes treated with high (25 mM) glucose concentration. Hyperglycemia reduced insulin-induced glucose uptake by these cells which was associated with reduced PI3K activity and higher activity of lipid phosphates PTEN ultimately leading to lower phosphoinositide 3′,4′,5′-triphosphate (PIP_3_). In addition, hyperglycemia decreased tyrosine phosphorylation of the insulin receptor substrate-1 (IRS-1) as well as phosphorylation of protein kinase Akt and the expression of insulin-sensitive glucose transporter GLUT4. All these effects of hyperglycemia were abolished by H_2_S as well as by l-cysteine. The protective effect of l-cysteine was blocked by CSE inhibitor PAG suggesting the involvement of H_2_S. Neither H_2_S nor l-cysteine had any effect on glucose uptake by cells cultured at normoglycemic conditions. Furthermore, the same group [54] has demonstrated that hyperglycemia decreased CSE expression and H_2_S production by 3T3-L1 cells indicating that hyperglycemia-induced insulin resistance could be mediated by the downregulation of CSE-H_2_S pathway.

Vitamin D intake and blood concentrations of vitamin D metabolites are inversely correlated with insulin sensitivity. In addition, vitamin D supplementation at low doses is suggested as the potential therapeutic approach in type 2 diabetes mellitus. Interestingly, Manna et al. [54] have demonstrated that the active metabolite of vitamin D_3_, 1,25-dihydroxycholecalcipherol (1,25-(OH)_2_-D_3_), increases CSE expression and H_2_S production by 3T3-L1 adipocytes cultured in high glucose conditions. In addition, 1,25-(OH)_2_-D_3_ improved insulin signaling (IRS-1 phosphorylation, PI3K activity and Akt phosphorylation) and increased insulin-stimulated glucose uptake, and these effects were abolished by CSE inhibition or knockdown. These results suggest that H_2_S may be involved in beneficial effect of vitamin D on insulin sensitivity.

Similarly, Xue et al. [55] have demonstrated that NaHS at 25 μM and 50 μM increased glucose uptake by 3T3-L1 adipocytes in the presence (but not in the absence) of insulin by 40% and 70%, respectively. The effect was observed in cells cultured at normo- or hyperglycemic conditions and was reproduced by H_2_S gas solution. H_2_S increased several components of the insulin signaling cascade that is phosphorylation of the insulin receptor β-subunit, PI3K activity and Akt phosphorylation. Interestingly, in the cell-free system NaHS increased insulin receptor tyrosine phosphorylation and its kinase activity suggesting that insulin receptor may be the direct target for stimulatory effect of H_2_S, possibly by cysteine persulfidation. Insulin receptor and/or IRS-1 may be dephosphorylated and inactivated by protein tyrosine phosphatase 1B (PTP1B). Although H_2_S has been demonstrated to inhibit PTP1B in other systems [56], this was not observed in 3T3-L1 cells [55]. In vivo, NaHS administered at a dose of 30 μmol/kg/day increased systemic insulin sensitivity in Goto-Kakizaki rats, the experimental animal model of type 2 diabetes; the effect accompanied by up-regulation of PI3K and Akt phosphorylation in the adipose tissue [55].

In mice fed regular chow, GYY4137 impaired insulin sensitivity as evidenced by the increase in plasma glucose and insulin concentrations as well as HOMA-IR index, the marker of insulin resistance [46]. Although PAG had no effect on fasting glucose and insulin concentrations, it slightly improved glucose tolerance as evidenced by lower area-under the curve of plasma glucose concentration in oral glucose tolerance test. These results suggest that H_2_S decreases insulin sensitivity in control mice. In contrast, GYY4137 reduced plasma glucose and insulin levels as well as HOMA-IR index and reduced area under the curve of plasma glucose concentration in the glucose tolerance test in mice receiving high fat diet for 13 weeks [46]. Surprisingly, PAG had similar effect to GYY4137, however, this should be treated with caution due to limited specificity of this inhibitor. Overall, these results suggest that H_2_S impairs insulin sensitivity in lean animals but has the opposite effect in high fat diet obese ones. Nevertheless, it is unclear how much of the effect on insulin sensitivity in that study was mediated by the adipose tissue [46].

## 7. Role of H_2_S in Obesity-Associated Adipose Tissue Expansion

Cai et al. [45] have recently demonstrated that treatment of high fat diet fed mice with H_2_S solution or GYY4137 (both at 100 μmol/kg/day) increased body weight, epididymal, perirenal and subcutaneous adipose tissue depots. In contrast, treatment of these mice with PAG at 30 mg/kg/day had the opposite effect [46]. This effect of H_2_S on adipose tissue could result from stimulation of adipogenesis, anabolic effect of insulin and triglyceride storage. Indeed, H_2_S and GYY4137 increased whereas PAG reduced total DNA content in the adipose tissue (the marker of cell number) as well as the expression and persulfidation of PPAR-γ but simultaneously, increased the average adipocyte size. These results indicate that H_2_S stimulates both adipogenesis and adipocyte hypertrophy. Interestingly, H_2_S or GYY4137 increased insulin sensitivity of high fat diet fed mice as evidenced by greater glucose tolerance and reduced insulin tolerance. Overall, the effect of H_2_S/GYY4137 in high fat diet fed mice resembles that of PPAR-γ agonists, thiazolidinediones, which increase insulin sensitivity by stimulating adopogenesis and increasing formation of small more insulin-sensitive adipocytes, but simultaneously may induce body weight gain.

## 8. Hydrogen Sulfide in Perivascular Adipose Tissue

Perivascular adipose tissue (PVAT) is the specific visceral adipose tissue depot which surrounds most of large and medium-sized arteries and also some veins. Initially considered only as the passive component of the vascular wall, PVAT appears to be involved in the regulation of vascular homeostasis. Under physiological conditions PVAT produces several mediators which reduce vascular tone, inhibit smooth muscle cell proliferation and suppress inflammatory reaction such as nitric oxide, adrenomedullin and angiotensin (1–7). In contrast, in pathological conditions such as arterial hypertension, obesity and type 2 diabetes PVAT becomes dysfunctional. Production of the abovementioned “beneficial” mediators is impaired and PVAT starts to produce vasoconstricting, proinflammatory and growth-promoting factors such as reactive oxygen species, proinflammatory cyto- and chemokines and growth factors [57,58,59]. It was first reported in 2009 that rat periaortic adipose tissue (PAT) expresses CSE and produces H_2_S in the presence of l-cysteine and pyridoxal 5′-phosphate. Contractility of aortic rings in response to several vasoconstrictors such as 5-hydroxytryptamine (5-HT), phenylephrine and angiotensin II was higher if PAT was maintained than when PAT was removed from the preparation. This anti-contractile effect of PAT was attenuated by propargylglycine and glibenclamide and was mimicked by exogenous H_2_S as well as by l-cysteine and pyridoxal 5′-phosphate. These results indicated that H_2_S is one of the adipose tissue-relaxing factors involved in the regulation of vascular tone [60].

Subsequently, Schleifenbaum et al. [61] have demonstrated that PVAT reduces contractility of rat mesenteric arteries which represent smaller-size peripheral arteries more relevant for the regulation of total peripheral resistance than large conduit arteries such as aorta. The anticontractile effect of PVAT on mesenteric arteries was also abolished by PAG and mimicked by NaHS, however, was not affected by glibenclamide suggesting that in contrast to aorta, K_ATP_ channels are not involved. In contrast, anticontractile effect of PVAT or NaHS on rat mesenteric arteries was attenuated by voltage-sensitive K^+^ channels (Kv) inhibitor, 4-aminopyridine (4-AP) as well as by the specific Kv7.x (KCNQ) channel inhibitor, XE991, and was mimicked by KCNQ channel activator, retigabine. Retigabine relaxed mesenteric arteries without adipose tissue as well as arteries with preserved adipose tissue but with CSE inhibited by PAG. Thus, the precise molecular target for PVAT-derived H_2_S in smooth muscle cells differs depending on the vessel size. However, Kohn et al. [62] demonstrated that both glibenclamide and XE991 blocked the anticontractile effect of PVAT on rat aortic rings as well as anticontractile effect of NaHS on PVAT-denuded aortic rings suggesting that both K_ATP_ and KCNQ channels may be required for full relaxing effect of H_2_S. Interestingly, the role of H_2_S may be species-specific. Indeed, the expression of CSE is much lower in mice than in rat periaortic adipose tissue, and although PVAT reduces contractility of mouse aortic rings, this effect is not mediated by H_2_S [62].

H_2_S production by periaortic adipose tissue is stimulated by phenylephrine, 5-HT and angiotensin II [61]. In addition, experimental hypertension induced by constriction of abdominal aorta up-regulates the expression of CSE and increases H_2_S production by periaortic adipose tissue. Aging is associated with progressive increase in CSE expression and decrease in H_2_S production [61]. The mechanism of the dissociation between CSE expression and H_2_S production in aging animals is unclear.

Several studies have demonstrated that obesity is associated with impaired anticontractile or even paradoxical procontractile effect of perivascular adipose tissues in animals and humans [63,64,65,66]. To understand the role of H_2_S in perivascular adipose tissue dysfunction, we examined the effect of experimental obesity induced by high-calorie diet on anticontractile effect of perivascular adipose tissue on the contractility of aortic rings in the rat. We found that the effect of obesity was dependent on time and composition of high-calorie diet. In rats fed high calorie diet for 3 months irrespectively of diet composition (that is both normal-fat and high-fat intake), anticontractile effect of PVAT was impaired which was associated with lower expression and activity of CSE as well as reduced H_2_S production by isolated PVAT ex vivo. Rats made obese by feeding high-calorie diet for 3 months were characterized by hyperlipidemia and insulin resistance. Treating these animals with PPAR-γ agonists, rosiglitazone, did not reduce body weight or PVAT depot size but increased insulin sensitivity, increased CSE expression/activity and H_2_S production in PVAT and restored anticontractile effect of PVAT on aortic rings [67]. Thus, high calorie diet-induced insulin resistance and/or hyperinsulinemia seem to downregulate the CSE-H_2_S pathway in PVAT. In contrast, anticontractile effect of PVAT was enhanced in animals fed high-calorie diet for 1 month. These animals had normal plasma lipid profile, insulin concentration and insulin sensitivity as well as unchanged CSE expression, however, H_2_S production by PVAT measured ex vivo was higher than in control animals. Furthermore, we estimated mitochondrial H_2_S oxidation in PVAT by measuring net H_2_S production in the absence and in the presence of SQR inhibitor, stigmatellin. H_2_S production in the presence of stigmatellin was higher than in the absence of this inhibitor. H_2_S production measured in the presence of stigmatellin was similar in lean and obese rats, however, mitochondrial H_2_S oxidation calculated as the difference in its production in PVAT incubated in the presence and in the absence of stigmatellin was reduced in obese rats [68,69]. The mechanism through which short-term obesity impairs mitochondrial H_2_S oxidation seems to be different depending on the diet. In rats fed normal-fat highly-palatable diet, the amount of mitochondria in PVAT was normal. In addition, the activity of SQR at saturating H_2_S and coenzyme Q concentration as well as mitochondrial function measured as the mitochondrial inner membrane potential were similar to control rats. Nevertheless, oxygen pressure was markedly reduced within the PVAT in obese animals, consistently with many studies indicating that obesity is associated with adipose tissue hypoxia due to greater diameter of adipocytes and reduced relative density of capillaries [70]. Thus, hypoxia is most likely responsible for the impairment of mitochondrial H_2_S oxidation. H_2_S has been suggested to operate as the oxygen sensor in some systems such as blood vessels, carotid body chemoreceptors and the kidney [71]. According to this theory, H_2_S is synthesized at relatively constant rate but under normal oxygenation is rapidly metabolized in mitochondria. When pO_2_ decreases substantially, H_2_S oxidation is impaired, its local concentration increases and signaling is augmented. Adipose tissue could be another organ in which H_2_S operates as the oxygen sensor.

In contrast, in rats fed high-fat diet for 1 month, the amount of mitochondria in PVAT was reduced as evidenced by lower concentration of mitochondrial DNA, cytochrome c concentration and citrate synthase activity. In addition, the expression of transcription factors involved in mitochondrial biogenesis, PPAR-γ coactivator-1α (PGC-1α), nuclear respiratory factor-1 (NRF-1) and mitochondrial transcription factor A (TTFAM) was also reduced [68,69]. These results suggest that short-term feeding with high fat diet impairs H_2_S oxidation in the adipose tissue by suppressing mitochondrial biogenesis. Previously, it has been demonstrated that natural or synthetic cannabinoids impair mitochondrial biogenesis in subcutaneous adipose tissue whereas cannabinoid CB_1_ receptor antagonist, rimonabant, has the opposite effect [72,73]. Endogenous cannabinoid system is active in the adipose tissue as evidenced by the expression of endocannabinoid synthesizing and metabolizing enzymes as well as cannabinoid receptors [74]. Therefore, we examined if endogenous cannabinoid system (ECS) is involved in obesity-induced impairment of mitochondrial biogenesis. For this purpose, the separate group of high fat diet-fed rats was treated with cannabinoid CB_1_ receptor antagonist, rimonabant (10 mg/kg/day), for the last 2 weeks of high fat diet application. Rimonabant reduced net H_2_S production by isolated PVAT slices, increased the amount of mitochondria and the expression of PGC-1α, NRF-1 and TFAM. In addition, concentration of endocannabinoid 2-arachidonylglycerol in PVAT was higher in high fat diet fed than in control animals. The expression of CB_1_ receptor at the mRNA level was significantly higher and its expression at the protein level tended to be higher (although not significantly) in PVAT of high fat-fed than in lean control rats. Taken together, these results indicate that short-term feeding with the high fat diet up-regulates endocannabinoid system in perivascular adipose tissue leading to suppression of mitochondrial biogenesis, impairment of H_2_S oxidation and augmentation of H_2_S-mediated anticontractile effect of PVAT.

Recently, Emilova et al. [75] have demonstrated that strepozotocin-induced diabetes, the model of type 1 diabetes, impairs anticontractile PAG-sensitive effect of PVAT in rat gracilis artery. Interestingly, at highest concentration of vasoconstrictor (5-HT) PVAT even enhanced vasoconstriction in diabetic rats whereas PAG exhibited relaxing effect. These results indicate that H_2_S-mediated vasodilatory effect of PVAT is not only impaired in diabetes but H_2_S may become vasoconstricting.

## 9. Hydrogen Sulfide and Dysregulation of Adipokine Production and Adipose Tissue Inflammation

Obesity and diabetes are associated with dysregulation of adipokine production as well as adipose tissue inflammation. Until now, the role of H_2_S in this context has not been studied extensively. It has been demonstrated that high glucose concentration (25 mM) decreases CSE expression at the mRNA and protein levels in 3T3-L1 adipocytes which is associated with up-regulation of MCP-1 and down-regulation of adiponectin [76]. In addition, forced CSE overexpression or NaHS treatment reduced MCP-1 production and increased adiponectin synthesis by these cells. These data indicate that detrimental effect of hyperglycemia on adipokine profile may be mediated, at least in part, by H_2_S deficiency in the adipose tissue.

Obesity is associated with accumulation of macrophages in the adipose tissue and their shift from antiinflammatory (M_2)_ to proinflammatory (M_1_) phenotype. M_2_ to M_1_ switch is mediated by opening of plasma membrane store-operated calcium channels and increase in intracellular Ca^2+^ concentration. Adipose tissue macrophages isolated from mice with diet-induced obesity are characterized by lower intracellular H_2_S concentration despite greater CSE expression and activity. Lipolysaccharide (LPS) increases the expression of CSE but reduces intracellular H_2_S concentration in cultured RAW264.7 macrophages indicating that inflammation is associated with H_2_S deficiency most likely due to its increased consumption/metabolism. In addition, LPS stimulated store-operated calcium entry (SOCE). Whereas GYY4137 had no effect on SOCE under baseline conditions, it reduced SOCE in LPS-stimulated cells. Moreover, PAG and aminooxyacetate as well as CSE silencing by the specific siRNA reduced intracellular H_2_S and stimulated SOCE response in RAW264.7 cells as well as in primary mice macrophages. Increase in M_1_ markers such as IL-6, IL-1β, and inducible nitric oxide synthase and decrease in M_2_ markers such as arginase-2 or mannose receptor C type 1 (MRC1) in obese mice are reversed by GYY4137 as well as SOCE inhibitors. Overall, these results indicate that H_2_S deficiency contributes to SOCE activation and proinflammatory phenotype switch of adipose tissue macrophages associated with obesity [77].

## 10. Adipose Tissue H_2_S as a Target for Pharmacotherapy

Beneficial effects of PVAT-derived H_2_S on vascular tone as well as its antiinflammatory and insulin-sensitizing properties demonstrated in other adipose tissue depots suggest that increasing H_2_S signaling could be the potential new therapeutic strategy for the treatment of obesity and metabolic syndrome. However, H_2_S donors currently used in research suffer from many important disadvantages. Inorganic sulfide salts such as NaHS or Na_2_S increase H_2_S concentration rapidly and for the short time. Supraphysiological and even toxic concentration of H_2_S in solution may appear after application of these salts. In addition, sulfide may be spontaneously oxidized to other sulfur species with differing physiological properties in the uncontrolled manner [78]. Some organic slowly-releasing H_2_S donors such as GYY4137 or H_2_S-releasing antiinflammatory drugs have become available but they may possess H_2_S-independent activities mediated by the parent compound.

Another approach is to modify H_2_S concentration and/or signaling by some of the currently used drugs. For example, zofenopril, one of the angiotensin converting enzyme (ACE) inhibitors currently used in the clinical practice is a H_2_S donor and exerts some activities in the cardiovascular system independent of ACE-inhibiting properties [79]. Statins are competitive inhibitors of 3-hydroxy-3-methylglutarylcoenzyme A reductase, the rate-limiting enzyme in cholesterol biosynthesis, and are commonly used in the prevention and treatment of atherosclerosis. Statins not only reduce blood LDL-cholesterol concentration but also exhibit so called “pleiotropic” activities resulting from the inhibition of various active products of the cholesterol biosynthesis pathway. We examined the effect of statins on H_2_S system in perivascular adipose tissue in the rat. We have demonstrated that lipophilic atorvastatin but not hydrophilic pravastatin increased the anticontractile effect of PVAT on rat aortic rings [80]. This effect resulted from the decrease in coenzyme Q (CoQ) which is the electron acceptor in the reaction of H_2_S oxidation by sulfide:quinone oxidoreductase. Indeed, atorvastatin had no effect on CSE expression and activity in PVAT but reduced mitochondrial oxidation of this gasotransmitter by decreasing CoQ concentration. CoQ supplementation either in vitro or in vivo normalized H_2_S production and reduced anticontractile effect of PVAT in atorvastatin-treated rats. Pravastatin had no effect on CoQ concentration and H_2_S oxidation in PVAT, most likely due to its hydrophilicity and limited penetration to the adipose tissue [80].

Adenosine and guanosine phosphorothioates (AMPS and GMPS) are synthetic AMP and GMP derivatives with one oxygen atom replaced by sulfur. Both these compounds may be hydrolyzed to H_2_S and AMP or GMP, respectively, by intracellular histidine triad-containing Hint-1 and Hint-2 proteins [81,82]. Although AMPS and GMPS do not permeate plasma membranes, they may enter the cells through purinergic P2X_7_ receptors/channels [83]. Recently we have demonstrated that AMPS and GMPS induce vasorelaxation of rat aortic rings with intact PVAT if P2X_7_ agonist is simultaneously added to the medium [84]. In contrast, AMPS and GMPS are not converted to H_2_S by aortic rings without PVAT, presumably because they cannot enter to vascular smooth muscle cells which do not express P2X_7_. Interestingly, AMPS and GMPS are converted to H_2_S by PVAT slices isolated from obese rats even without synthetic P2X_7_ agonist, probably because these channels are overexpressed and/or activated in adipose tissue of obese animals [84]. These nucleotide phosphorothioates possess several advantages as the potential H_2_S donors. First, they are derivatives of natural compounds (AMP and GMP) which enter normal metabolic pathways and as such are not toxic. AMPS and/or AMP originating from it may additionally activate AMP-stimulated protein kinase (AMPK) potentially offering additional metabolic benefits. Second, AMPS and GMPS release H_2_S intracellularly and therefore may be quantitatively more efficient as the H_2_S donors than compounds which release H_2_S in the non-enzymatic uncontrolled manner. Finally, at least theoretically, it could be possible to control AMPS and GMPS entry to the cells or possibly their enzymatic hydrolysis. In particular, as P2X_7_ are activated during the inflammation, AMPS and GMPS could be more specifically delivered to the injured or inflamed tissues such as adipose tissue of obese humans.

Organosulfur compounds contained in garlic such as S-allylcysteine, diallyl disulfide (DADS) or diallyl trisulfide (DATS) may release H_2_S in the cells in the presence of reducing compounds. It is suggested that some beneficial effects of garlic preparations may be mediated by H_2_S [85]. Interestingly, garlic polysulfides have been demonstrated to increase the amount of brown adipose tissue, stimulate energy expenditure and consequently reduce white adipose tissue depots and improve insulin sensitivity in animals and humans [86,87]. However, it is unclear if and how much of these effects are mediated by H_2_S because other products of garlic polysuflide degradation could have the independent activities.

## 11. Sulfur Dioxide—Another Sulfur Gasotransmitter in the Adipose Tissue?

Recent studies suggest that H_2_S, the most reduced sulfur compound, is only one of reactive sulfur species (RSS) involved in cell signaling [78]. More oxidized sulfur species such as inorganic polysulfides (H_2_S_n_) as well as organic sulfane sulfur compounds such as cysteine or glutathione hydropersulfides (Cys-SSH, GSSH) may be responsible for some biological effects of H_2_S. In addition, sulfur dioxide (SO_2_)/sulfurous acid (H_2_SO_3_) and its dissociation products, bisulfite (HSO_3_^−^) and sulfite (SO_3_^2−^) may be the important inorganic signaling molecules [88]. SO_2_ is synthetized during H_2_S oxidation pathway by sulfur dioxygenase and is rapidly oxidized to sulfate by sulfite oxidase [11]. In addition, sulfur dioxide/sulfite may be formed during spontaneous reaction of superoxide produced by neutrophils with H_2_S. Alternatively, sulfur dioxide/sulfite is produced from l-cysteine by sequential activities of cysteine dioxygenase, and aspartate aminotransferase (AST) with cysteine sulfinate and β-sulfinpyruvate as the intermediates [88]. Both SO_2_ and SO_2_-producing enzyme, AST1, are detected in perivascular, perirenal, epididymal and subcutaneous white adipose tissue as well as in brown adipose tissue of the rat [89]. Exogenous SO_2_ or overexpression of AST1 inhibit TNF-α-stimulated production of MCP-1 and IL-8 by 3T3-L1 adipocytes. In contrast, knockdown of AST1 reduced SO_2_ production from l-cysteine and enhanced TNF-α-induced secretion of MCP-1 and IL-8, as well as increased the activity of proinflammatory transcription factor, NFkB, in these cells. The effect of AST1 knockdown was reversed by SO_2_. These results indicate that SO_2_ produced in the adipose tissue may inhibit adipose tissue inflammation associated with obesity.

## 12. Conclusions and Future Perspectives

Hydrogen sulfide is synthesized in adipose tissue and is involved in the regulation of adipogenesis, metabolism and adipokine production. Although significant progress has been made in this field during the recent years, much work is still to be done. Some effects of H_2_S in adipose tissue such as its role in the regulation of insulin sensitivity and lipolysis are controversial. The effect of obesity/metabolic syndrome on H_2_S system in the adipose tissue is dependent on diet composition, time of high calorie feeding and specific adipose tissue depot. The mechanism of this effect is complex and incompletely understood. Due to these controversies, the perspectives of using H_2_S donors or augmenting H_2_S signaling to improve adipose tissue dysfunction in common metabolic pathologies should be treated with caution. Nevertheless, the majority of studies suggest that augmenting H_2_S signaling could reduce adipose tissue inflammation and correct related metabolic abnormalities.
